# Shifts in the Abundances of Saprotrophic and Ectomycorrhizal Fungi With Altered Leaf Litter Inputs

**DOI:** 10.3389/fpls.2021.682142

**Published:** 2021-07-21

**Authors:** Sara Marañón-Jiménez, Dajana Radujković, Erik Verbruggen, Oriol Grau, Matthias Cuntz, Josep Peñuelas, Andreas Richter, Marion Schrumpf, Corinna Rebmann

**Affiliations:** ^1^Center for Ecological Research and Forestry Applications (CREAF), Bellaterra, Spain; ^2^Spanish National Research Council (CSIC), Global Ecology Unit CREAF-CSIC-UAB, Bellaterra, Spain; ^3^Centre of Excellence Plant and Ecosystems (PLECO), Department of Biology, University of Antwerp, Antwerp, Belgium; ^4^French Agricultural Research Centre for International Development (CIRAD), Joint Research Unit Ecology of Guianan Forests-UMR EcoFoG (AgroParisTech, CNRS, INRA, University of Antilles, University of Guyane), Kourou, French Guiana; ^5^Université de Lorraine, French National Institute of Agricultural Research, AgroParisTech, UMR 1434 Silva, Nancy, France; ^6^Department of Microbiology and Ecosystem Science, University of Vienna, Wien, Austria; ^7^Department for Biogeochemical Processes, Max Planck Institute for Biogeochemistry, Jena, Germany; ^8^UFZ—Helmholtz Centre for Environmental Research, Department of Computational Hydrosystems, Leipzig, Germany

**Keywords:** CO_2_ fluxes, Gadgil effect, ectomycorrhiza fungal exploration types, litter decomposition, soil fungal communities, plant detritus

## Abstract

Ectomycorrhizal (EcM) and saprotrophic fungi interact in the breakdown of organic matter, but the mechanisms underlying the EcM role on organic matter decomposition are not totally clear. We hypothesized that the ecological relations between EcM and saprotroph fungi are modulated by resources availability and accessibility, determining decomposition rates. We manipulated the amount of leaf litter inputs (No-Litter, Control Litter, Doubled Litter) on Trenched (root exclusion) and Non-Trenched plots (with roots) in a temperate deciduous forest of EcM-associated trees. Resultant shifts in soil fungal communities were determined by phospholipid fatty acids and DNA sequencing after 3 years, and CO_2_ fluxes were measured throughout this period. Different levels of leaf litter inputs generated a gradient of organic substrate availability and accessibility, altering the composition and ecological relations between EcM and saprotroph fungal communities. EcM fungi dominated at low levels of fresh organic substrates and lower organic matter quality, where short-distances exploration types seem to be better competitors, whereas saprotrophs and longer exploration types of EcM fungi tended to dominate at high levels of leaf litter inputs, where labile organic substrates were easily accessible. We were, however, not able to detect unequivocal signs of competition between these fungal groups for common resources. These results point to the relevance of substrate quality and availability as key factors determining the role of EcM and saprotroph fungi on litter and soil organic matter decay and represent a path forward on the capacity of organic matter decomposition of different exploration types of EcM fungi.

## Introduction

Plants adjust the amount of carbon (C) invested in above- vs. belowground parts in response to soil nutrient availability to optimize biomass returns of invested carbon (Shipley and Meziane, 2002; Dybzinski et al., 2011). Accordingly, plants have been shown to allocate more C to mycorrhizal fungi in exchange for nutrients at elevated CO_2_ and lower nutrient availability (Treseder, 2004; Alberton et al., 2005; Högberg et al., 2010; Phillips et al., 2011). Shifts in the amount of above- and belowground organic matter inputs to the soil may also shape the composition and activity of fungal communities (Yarwood et al., 2009; de Graaff et al., 2010; Kaiser et al., 2010), with potential implications for decomposition rates of organic matter and soil C storage.

EcM and saprotrophic fungi interact in the breakdown of litter-derived organic substrates, determining the decomposition rates, the fate and the stabilization of soil organic matter (SOM) (Lindahl and Tunlid, 2015). Many EcM fungi have the ability to produce oxidative enzymes for the breakdown of organic compounds (Bödeker et al., 2009; Nicolás et al., 2019), which can alter the degradability of residual SOM by the saprotroph community (Rineau et al., 2012). However, EcM fungi may also compete with the saprotrophic community for limiting water, nutrients or space (Lindahl et al., 2001; Koide and Wu, 2003; Bödeker et al., 2016). This competition has been long hypothesized to slow down decomposition rates (i.e., “Gadgil effect,” Gadgil and Gadgil, 1971, 1975), leading to the prediction of larger C storage in forest soils (Orwin et al., 2011; Averill et al., 2014). Nonetheless, EcM fungi have shown both no effect (Mayor and Henkel, 2006; but see McGuire et al., 2010) and a stimulation of decomposition rates (Entry et al., 1991; Zhu and Ehrenfeld, 1996; Brzostek et al., 2015) and the mechanisms underlying the EcM role on organic matter decomposition are not totally clear (Fernandez and Kennedy, 2015).

Despite sharing common ancestors (Tedersoo et al., 2010), saprotrophs and ectomycorrhiza fungi have evolved into two fungal groups with well-differentiated resource needs and acquisition strategies. Some EcM guilds have retained the capacity to breakdown organic nitrogen (N) substrates (Shah et al., 2016; Nicolás et al., 2019) and can exploit litter selectively for N (Rineau et al., 2012; Bödeker et al., 2014). The direct supply of C from the plant host may confer an advantage to EcM fungi when organic substrates are not easily available, allowing them to allocate more resources to exploit and acquire N (Rineau et al., 2013; Lindahl and Tunlid, 2015). In line with this, fungal communities have shown segregated niche distribution with depth, where saprotrophs dominate in organic and cellulose-rich litter layers and EcM fungi are more abundant at greater depths, where the acquisition of N from more recalcitrant organic substrates also requires higher energy investment (Lindahl et al., 2007; Baldrian et al., 2012). Saprotrophic decomposition may be progressively restricted as the organic substrates become less accessible and the energy return of their decomposition declines (Baldrian, 2009; Sterkenburg et al., 2018). This niche segregation may be then the consequence of the specialization of each fungal group on divergent target resources and the development of different evolutionary strategies for their acquisition. Changes in substrates quality and availability can, therefore, drive shifts in the composition and abundance of EcM and saprotrophic fungi thereby modulating their ecological interactions and the decomposition rates of organic matter as a result.

In order to elucidate how resource availability govern the ecological feedbacks between saprotrophs and EcM fungal communities, we manipulated the amount of leaf litter inputs (No-Litter “NL,” Control Litter “CL,” Doubled Litter “DL”) on Trenched (“T,” root exclusion) and Non-Trenched plots (“NT,” with roots) of a temperate deciduous forest composed of ectomycorrhiza-associated species (oak and beech). Resultant shifts in the diversity and composition of soil fungal communities as detected by ITS1 metabarconding and phospholipid fatty acids (PLFAs) and in the microbial biomass, dissolved organic C, and C and N stocks were determined after 3 years, and CO_2_ fluxes were measured throughout this period. We hypothesized that: (1) Changes in the availability and accessibility of organic substrates in soil in response to altered leaf litter inputs modulate the dominance and ecological interactions between EcM and saprotroph fungi; (2) Conditions of limited access to organic substrates will benefit EcM over saprotroph fungi, leading to exacerbated competition between both groups for N sources, while plentiful and easily accessible organic matter inputs will benefit the proliferation of saprotrophs and relax the competition; (3) The exclusion of EcM fungi in Trenched plots will, therefore, increase saprotrophic abundance and decomposition rates in a larger extent at low litter inputs, resulting in a higher increase in the litter-derived CO_2_ flux at “Control Litter” inputs compared to “Doubled Litter” inputs.

## Materials and Methods

### Study Site

The study was conducted in a mixed deciduous forest (“Hohes Holz”) in the area of the Magdeburger Boerde in central Germany (52°05′N, 11°13′E, 210 m above sea level). Climate in the study area is subatlantic-submontane. Mean annual temperature is 9.1°C (climatic period 1981–2010, station Ummendorf of German Weather Service), with mean minimum temperature in the coldest month (January) of 0.7°C, and mean maximum of the warmest month (July) of 18.3°C. Annual mean precipitation was 563 mm during the climatic period 1981–2010, while annual precipitation during the experiment measured locally at the site was 550 mm in 2015 and 390 mm in 2016. The forest stand is located in a mainly municipal forest area, managed by regional forestry. The experiment was conducted inside a 1 ha fenced area with ungulate enclosure since 2011, composed of sessile oak [*Quercus petraea* (Matt.) Liebl.] and European beech (*Fagus sylvatica* L.) as the dominant species (45 and 38% of total basal area, respectively) with accompanying hornbeam (*Carpinus betulus* L., 13%) and birch (*Betula pendula* Roth, 4%). Tree height and diameter at breast height were 27.0 ± 11.9 (SD) m, and 0.38 ± 0.2 m on average for beech and oak. The bedrock is Pleistocene sandy loess above till and Mesozoic muschelkalk, with Haplic Cambisol as predominant soil type. Soil texture at 0–20 cm depth was 3.0 ± 1.8% sand, 87.1 ± 2.1% silt, and 10.0 ± 2.2% clay, with a pH of 8.0. The main soil variables determined before the experiment establishment are described in Table 1.

**Table 1:** Initial soil parameters measured prior to the establishment of the trenching and leaf litter input treatments.

**Soil depth (cm)**	**SOC (%)**	**TN (%)**	**C:N_**soil**_**	**Soil depth (cm)**	**ρ_soil_ (g cm^**−3**^)**
0–5	5.67 ± 0.39	0.335 ± 0.020	16.71 ± 0.26	0–5	1.15 ± 0.07
5–15	2.14 ± 0.17	0.121 ± 0.008	17.48 ± 0.33		
15–30	0.79 ± 0.05	0.051 ± 0.002	15.02 ± 0.38	20–25	1.35 ± 0.03
30–60	0.31 ± 0.02	0.035 ± 0.001	9.025 ± 0.41	40–45	1.49 ± 0.02
60–100	0.22 ± 0.01	0.032 ± 0.001	6.78 ± 0.27	60–65	1.55 ± 0.02

*SOC, soil organic carbon; TN, total soil nitrogen; C:N_soil_, soil C:N ratios; ρ_soil_, soil bulk density*.

### Experimental Design

**Figure 1:**
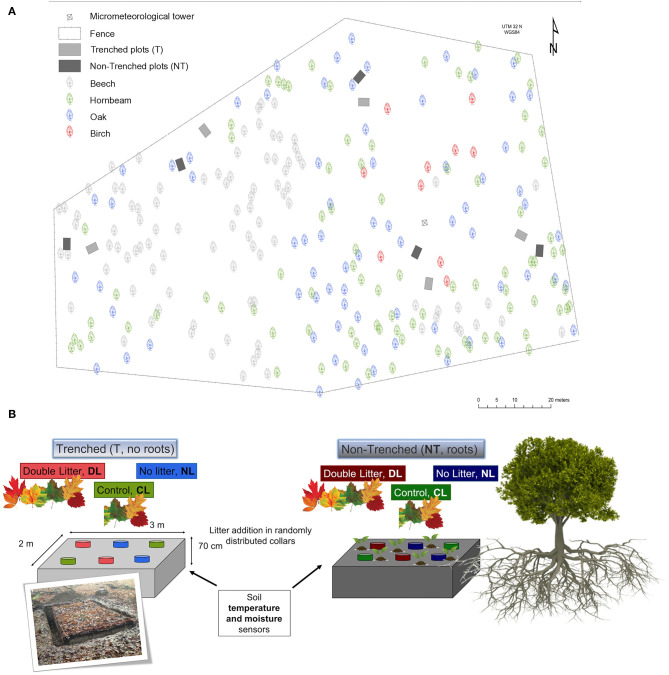
**(a)** Location of the paired Trenched and Non-Trenched plots in the study area. **(b)** Scheme of the experimental design showing the distribution of the trenching and leaf litter input treatments within each block.

Three levels of leaf litter inputs (No-Litter “NL,” Control Litter “CL,” Doubled Litter “DL”) were established in pairs of Trenched (“T,” root exclusion) and Non-Trenched plots (“NT,” with roots) following a factorial randomized block design. For this, five blocks were randomly distributed over 1 ha area, each one consisting of one trenched plot and a similar non-trenched plot of 2 × 3 m each, within a distance <10 m between them (Figure 1). The root exclusion in the Trenched plots was achieved by excavating a trench 20 cm wide and 70 cm depth around each plot in November 2013, 6 months before the start of the measurements. Trench walls were subsequently covered by a polyethylene foil and the trench was refilled back with soil in order to avoid the re-colonization of new roots. Soil remained unperturbed within the 2 × 3 m area of each plot. Understory vegetation was also removed in Trenched plots at the time of the experimental setting and every ca. 2 weeks during the growing season since the establishment of the experiment to exclude plant root contribution. Two 1 × 1 m replicate subplots per litter input level (NL, CL, DL) were randomly distributed in a 2 × 3 m grid within each plot in April 2014 (*n* = 5 blocks × 2 trenching levels (paired plots) × 3 l inputs levels × 2 replicate subplots = 60). Leaf litter was removed from No-Litter subplots (NL) and immediately added and distributed as evenly as possible to Doubled Litter subplots (DL) once a month and every 2 weeks during autumn. Litter was left undisturbed in the Control Litter subplots (CL), representing the litter amount that naturally falls over the forest floor (325 ± 49 g m^−2^ y^−1^). The distance from the center of each subplot to the nearest oak and beech tree were also measured.

### Soil CO_2_ Fluxes and Soil Environmental Variables

Prior to the establishment of litter input treatments in April 2014, a PVC collar (diameter 20 cm × height 11.5 cm) was inserted in the soil to ca. 5 cm depth in the center of each subplot. CO_2_ fluxes were measured on the inserted collars every 2 weeks from June 2014 to December 2016 using a portable dark, closed chamber connected to an automated CO_2_ analyzer system (Li-Cor 8100, Lincoln, NE, USA). Snow prevented the location of the PVC collars and CO_2_ measurements during winter. At each measuring campaign, CO_2_ fluxes were taken simultaneously with soil temperature at 10 cm depth using a probe thermometer (DET3R, Voltcraft, Wernberg-Köblitz, Germany), and with volumetric water content using a soil moisture probe (8100-204 Delta-T ThetaProbe, Cambridge, UK). Measurements were generally performed between 8 am to 4 pm. The order of measurement was rotated among the blocks and treatments over the campaigns. Vegetation cover was estimated visually from 0 to 100% for each campaign (Sutherland, 1996). Vegetation inside the collars was not removed in the case of Non-Trenched plots. Thus, CO_2_ fluxes reported in this study could include some above-ground autotrophic respiration. However, for practical proposes and because the latter are likely negligible, we use “soil CO_2_ fluxes” as shorthand for the CO_2_ flux measured at the soil surface, which are compounded of soil respiration and litter decomposition in case of DL and CL litter input levels.

### Soil Sampling

Three years after the experiment establishment (May 2017), samples of mineral soil were collected from a randomly chosen subplot per block, litter input and trenching level at two depths (0–5 cm and 5–10 cm) (*n* = 5 blocks × 2 trenching level × 3 l input × 2 depths = 60 soil samples). Soil samples were immediately sieved to 2 mm and homogenized. The bulk density was calculated with the dry weight and volume of the soil fraction <2 mm. The fraction of soil >2 mm (stones, hereafter) was also weighted. The root biomass in each soil sample was carefully washed and oven dried at 60°C until constant weight. The fresh sieved soil samples were split into three soil subsamples: A 50 g subsample of fresh soil was weighted, transferred to a paper bag and oven dried at 105°C for 48 h for gravimetric determination of water content by the difference between fresh and dry weight, and stored for elemental analyses of inorganic and organic soil C and total soil N. A second 50 g subsample from the upper 5 cm of soil was frozen at −20°C within 24 h after sampling for further analyses of fungal community composition by DNA sequencing and PLFAs analyses. The remaining fresh soil was immediately stored at 4°C for further analyses (see below).

### Litter Sampling

Litter samples were also collected from the same randomly chosen subplot per block, litter (only CL and DL) and trenching levels (*n* = 5 blocks × 2 trenching levels × 2 l input levels = 20 l samples) at the same time as the soil sampling (3 years after the experiment establishment, May 2017). For that, a 15 × 15 cm quadrant was placed randomly in each subplot and the total amount of litter and organic layer falling within the quadrant perimeter was collected. No roots were found growing in the litter layer. Litter samples were then oven dried at 60°C, weighted, ground and thereby homogenized prior to the C and N elemental analysis.

### Soil Extractions and Chemical Analyses

A 6 g subsample of fresh mineral soil was extracted with 30 ml of 0.05M K_2_SO_4_ within 24 h of soil sampling. Another 6 g subsample was fumigated with CHCl_3_ for 24 h in vacuum to release the nutrients from the microbial biomass (fumigation-extraction method; Jenkinson and Powlson, 1976), after which the soil was also extracted with 0.05 M K_2_SO_4_ as above. Dissolved organic C (DOC) in fumigated and non-fumigated K_2_SO_4_ extracts was determined with a vario TOC cube (Elementar Analysensysteme GmbH, Hanau) and microbial C (C_micro_) was determined from the difference of DOC between fumigated and non-fumigated subsamples. We were only interested in relative differences among treatments, so the concentrations in the microbial fraction presented here were not corrected for extraction efficiency.

Total soil inorganic C, organic C (SOC), total soil N (TN), total litter C, and total litter N were determined from dry and homogenized soil and litter samples by dry combustion with an Elemental Analyzer (VarioMax CN, Elementar Analysensysteme GmbH, Hanau, Germany for soil samples and Vario EL II, Elementar Analysensysteme GmbH, Hanau, Germany for litter samples). C:N ratios of soil (C:N_soil_) and leaf litter (C:N_soil_) were calculated on a mass basis. The relative accessibility of the DOC pool was calculated as its ratio to the SOC pool. All fractions are presented relative to dry mass.

### Phospholipids Fatty Acid Analyses

Soil samples from the upper 5 cm of soil were freeze-dried and total lipids were extracted with a mixture of chloroform, methanol and citrate buffer (1:2:0.8, v/v/v) and fractionated by solid-phase extraction on silica columns according to Gorka et al. (2019). Phospholipids were derivatized to methyl esters via alkaline methanolysis and dried under a constant stream of N_2_. Resulting fatty acid methyl esters were re-dissolved in iso-octane and identified and quantified on a gas chromatograph (Agilent 7890B GC; Santa Clara, CA, USA) coupled to a time-of-flight mass spectrometer (Pegasus HT; LECO corporation, Saint-Joseph, MI, USA) on a DB5 column (60 m × 0.25 mm × 0.25 μm). Bacterial fatty acid methyl esters (BAME CP mix, Supelco; 37 Component FAME mix, Supelco) were used as qualitative standards. Prior to methylation nonadecanoic acid (19:0) was added to samples and used as an internal standard for quantification.

PLFA 18:2ω6,9 was used as an indicator of fungal abundance (Olsson, 1999). Since the proportions of individual PLFA differ between different types of bacteria (Ratledge and Wilkinson, 1988) and we were not interested on the shifts of bacterial communities, we grouped the bacterial PLFA to produce less variable results and ease the interpretation (Frostegård and Bååth, 1996; Zelles, 1999). Nonetheless, shifts in bacterial communities were also explored in order to check potential effects on the abundances of saprotrophic and ectomycorrhizal fungi. Beta-hydroxil, cyclopropane, and branched chain PLFAs were considered bacterial PLFAs (Zelles, 1999; Ruess and Chamberlain, 2010). The ratio between fungal and bacterial PLFAs was calculated and considered an indicator of fungal to bacterial biomass ratio. Total PLFAs were considered as a further estimation of total microbial biomass.

### DNA Extraction and Sequencing

DNA was extracted from 0.25 to 0.35 g of the mineral soil samples using the DNeasy PowerSoil Kit (Qiagen, Venlo, the Netherlands) following the manufacturer's protocol. The first PCR was performed using ITS1f and ITS2 primers (Smith and Peay, 2014) with Illumina Nextera labels (Illumina Inc; San Diego, CA, USA) targeting fungal ITS1 region. The 25 μl reaction mixtures contained 2 μl of DNA template, 480 nM forward and reverse primers and 1X PCR buffer, 200 μM of each dNTPsand 1 U of Phusion High-Fidelity DNA polymerase (New England Biolabs, Ispwich, MA, USA). PCR conditions were as follows: initial denaturation step at 98°C for 60 s, followed by 35 cycles of: denaturation step at 98°C for 30 s, annealing at 55°C for 30 s, extension at 72°C for 30 s; and final extension step of 72°C for 10 min. The mixture for the second PCR contained 2.5 μl of 50 × diluted PCR product and 0.1 μM of dual barcoded primers with Illumina adapters. The conditions were: initial step at 98°C for 60 s, 12 cycles: at 98°C for 10 s, 63°C for 30 s, 72°C for 30 s; and 72°C for 5 min. Following the gel electrophoresis (1.5% agarose gel) successful amplicons were normalized and purified from PCR artifacts using the SequalPrep Normalization Plate Kit (ThermoFisher Scientific; Waltham, MA, USA). Samples were then pooled into a single library and purified using QIAquick Gel Extraction Kit (Qiagen; Venlo, the Netherlands). Subsequently, the library was quantified with qPCR (KAPA Library Quantification Kits, Kapa Biosystems, Wilmington, MA, USA) and sequenced on the Illumina MiSeq platform (Illumina Inc; San Diego, CA, USA) with 300 cycles for paired-end reads.

### Bioinformatic Analyses

The bioinformatics analyses were performed using the USEARCH software following the UPARSE pipeline (Edgar, 2013). The sequences were first trimmed to 250 bp, forward and reverse reads were merged and primers were removed. Quality filtering was performed using a maximum expected error threshold of 0.5. After dereplication and singleton removal the sequences were clustered into operational taxonomic units (OTUs) based on 97% similarity using the UPARSE-OTU algorithm (Edgar, 2013) yielding 810 OTUs in total. The original reads were then mapped to the OTUs with the identity threshold of 0.97. The sequence counts of all samples (abundances matrixes) were normalized by random subsampling to the minimal number of reads per sample (2,986 sequences, rarefaction) to avoid potential artifacts due to library size and lower the false discovery rates (Weiss et al., 2017). We calculated rarefied OTU accumulation curves (“vegan” R package; Oksanen et al., 2020) to explore the completeness of our sampling. While rarefaction curves indicated that additional sequencing depth would yield additional taxa in most samples, depth was relatively balanced among the treatments (Supplementary Figure 1A), so unlikely to bias the abundance patterns after subsampling to a common read number.

The OTUs were taxonomically assigned by comparing representative sequences to the UNITE database (Kõljalg et al., 2015) (release date 01.08.2015), using the BLAST algorithm with default settings. The OTUs were assigned to particular taxa by selecting the hits with the lowest E-value and with a minimum alignment length of 75 bp. OTUs were subsequently assigned to functional groups if their genus was successfully matched with one of the genera whose trophic type have been described in Tedersoo et al. (2014). If the trophic type at the genus level was unknown, trophic type was assigned at family level if more than 80% of genera within that family, containing at least four genera, belonged to the same trophic type. EcM fungi were further assigned to different exploration types (contact, short, short-medium, medium and long) based on the description of functional traits of EcM genera in Lilleskov et al. (2011). As a result, 46.3% of total OTUs were assigned to a trophic type and 98.2% of OTUs assigned to EcM fungi could be grouped into an exploration type, which are similar to the percentages found in global studies on fungal functional ecology (e.g., Tedersoo et al., 2014).

### Statistical Analyses

The effect of root and litter inputs on monthly soil CO_2_ fluxes, soil temperature and soil moisture and its variation along the time was tested by repeated measures ANOVAs split-plot design, with trenching and litter input as main fixed factors between subjects, and date as factor within subjects. The analysis was thus run using mean monthly values per each treatment combination and block, which allowed us to produce integrated data of soil respiration per month. The excess of CO_2_ flux caused by the addition the litter inputs (hereafter “litter-derived CO_2_ flux”) was calculated as the difference of CO_2_ fluxes between levels with litter inputs (DL and CL) and the correspondent litter exclusion level (NL) in Trenched (root exclusion) and Non-Trenched plots (root presence). Litter-derived CO_2_ fluxes, as calculated here, have previously been shown to be reliable indicators for long-term litter decomposability and litter carbon dynamics (Bowden et al., 1993; Aerts and Caluwe, 1997; Xiao et al., 2014). Nonetheless, these fluxes should be interpreted as metric of the effect (direct and indirect) of the litter addition on CO_2_ fluxes, and not as a strict measure of C released exclusively from the litter. The effect of trenching, leaf litter inputs and their interactions on the mean and CO_2_ excess flux was tested using two-ways ANOVAs, with trenching and leaf litter inputs as fixed factors. Differences among leaf litter inputs levels within each trenching level were further tested by *post-hoc* tests with Tukey correction for multiple testing.

The effect of trenching, leaf litter inputs, soil depth and their interactions on soil DOC, C_micro_, SOC, TN, C:N_soil_, ρ_soil_, and on root biomass was tested using three-ways ANOVAs, with trenching, leaf litter inputs and soil depth as fixed factors. The effect of trenching and leaf litter inputs was also tested on the same variables averaged across soil depths using two-ways ANOVAs, since the effect of soil depth was consistent across treatments (no interactions with the rest of factors). Differences among leaf litter inputs levels within each trenching level were further tested by *post-hoc* tests with Tukey correction for multiple testing.

We investigated the similarity of the soil fungal communities amongst root and leaf litter inputs treatments by non-metric multidimensional scaling (NMDS) ordinations with the read-abundance data using the Bray–Curtis index (“BiodiversityR” package; Kindt and Coe, 2005). For exploratory purposes and to check the robustness of the data, we also performed ordinations using non-rarefied data, giving similar results (Supplementary Figure 2). We calculated the relative abundance of the different functional groups (number of reads of a given functional group/total number of reads per sample) and the relative abundance of the different EcM exploration types (number of reads of a given EcM exploration type/total number of reads of identified EcM fungi per sample) to assess the effect of root and leaf litter inputs on the relative abundances of each group and to account for the variation in the relative abundance of different functional groups and EcM exploration types amongst the samples. We then used the “envfit” function (“vegan” R package; Oksanen et al., 2020) to fit the relative abundances of different functional groups and the environmental variables onto the NMDS ordination. Environmental variables were previously standardized.

The dissimilarity between pairs of samples of each root and leaf litter input treatment levels (i.e., beta-diversity) was calculated using the Bray-Curtis distances to the centroid (“vegdist” and “betadisper” functions from “vegan” R package), and the homogeneity of multivariate group of dispersions was then tested using multi-factor analyses of variance for dissimilarity matrices (“dissmfacw” function from “TraMineR” R package, Studer and Ritschard, 2016). Since the dispersion around centroids of soil fungal communities differed significantly among trenching levels (see Fungal community shifts in Results section), the effect of root and leaf litter treatments on the fungal community composition was tested using analyses of deviance for multivariate generalized linear models (“manyglm” R function from “mvabund” R package, Wang et al., 2012), with trenching and leaf litter inputs as fixed factors, block as a random factor and a negative binomial as a link function.

The effect of root and leaf litter inputs on leaf litter variables, PLFAs groups and on the relative abundances of saprotrophic and EcM fungi was tested by two-ways ANOVAs, with trenching and leaf litter inputs as fixed factors. The effect of each factor was further explored by one-way ANOVAs for each trenching and leaf litter input level. Similarly, the effect of leaf litter inputs on EcM exploration types in NT plots (root trenching suppressed virtually all EcM fungi) was tested by one-ways ANOVAs with leaf litter inputs as fixed factor. Correlations between the relative abundances of different fungal trophic types and EcM exploration types and the soil and environmental variables were also explored by Pearson correlations.

Variables were transformed when required to improve normality and homoscedasticity (Quinn and Keough, 2009). Statistical analyses and model construction were performed using JMP 13.0 (SAS Institute) and R v.3.4.3 (R Core Team, 2016). All results are presented as means ± standard errors.

## Results

### Soil Variables and Microbial Biomass

**Figure 2:**
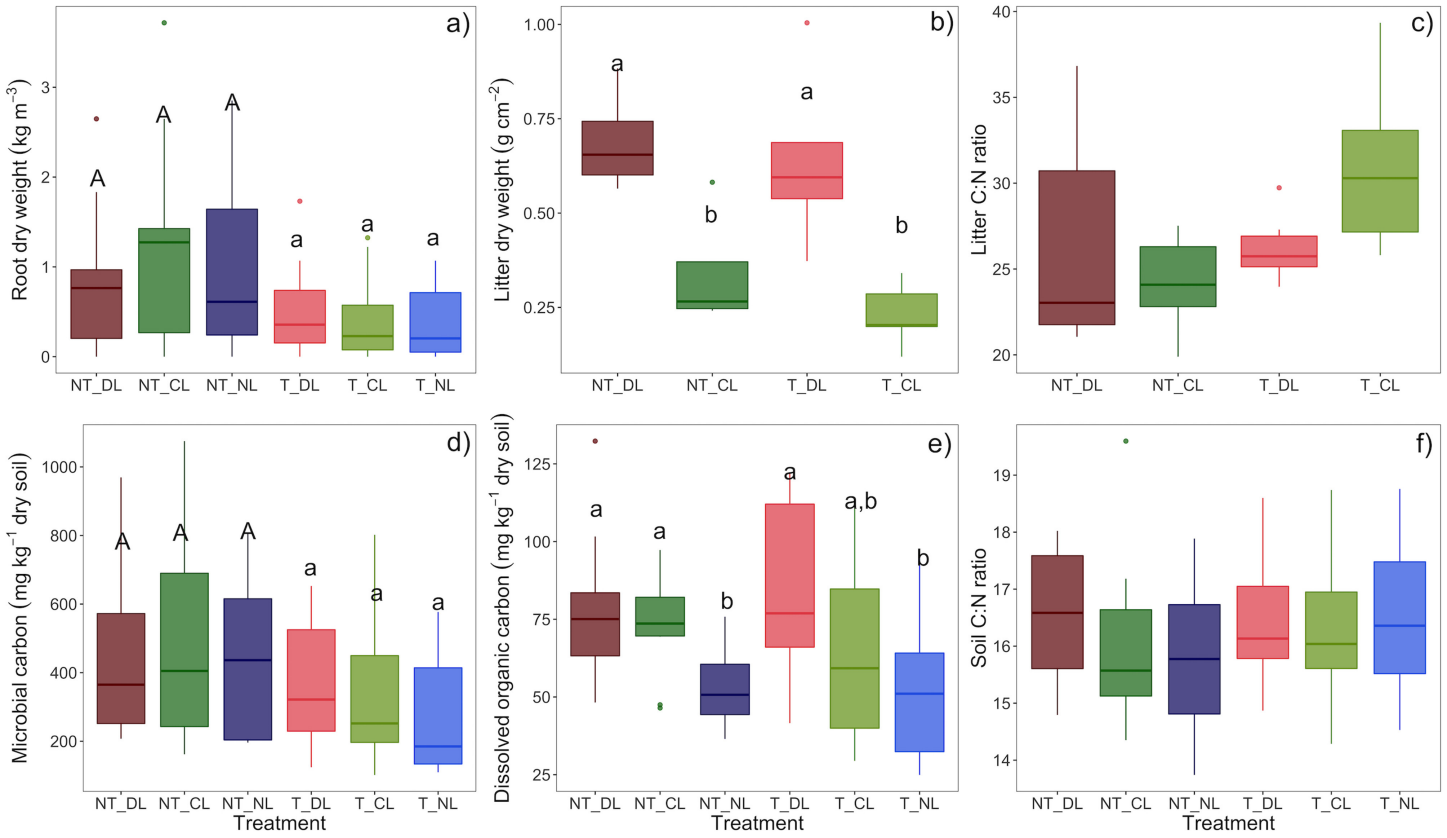
**(a)** Root biomass, **(b)** leaf litter biomass, **(c)** leaf litter C:N ratios, **(d)** microbial biomass carbon, **(e)** dissolved organic carbon, and **(f)** soil C:N ratios in each of the root and leaf litter input treatments. Averaged values of 0–5 and 5–10 cm soil depths for the case of root and soil variables. Box-plots represent the values of *n* = 5 plots per treatment. Different letter case indicates significant effect of trenching according two-ways ANOVA. Different letters indicate significant differences among leaf litter input levels within each trenching level. Microbial biomass carbon is not corrected for extraction efficiency. NT_DL, Non-Trenched and Doubled Litter; NT_CL, Non-Trenched and Control Litter; NT_NL, Non-Trenched and No-Litter; T_DL, Trenched and Doubled Litter; T_CL, Trenched and Control Litter; T_NL, Trenched and No-Litter.

Trenching reduced the amount of roots present in the soil, which was consistent across leaf litter input levels and depths (Table 2; Figure 2a). Leaf litter manipulation treatments also successfully altered the amount of leaf litter decomposing over the soil surface. As such, the dry weight of litter per unit of surface at the DL litter input treatment was approximately twice the amount of litter in the C litter input treatment both in T and NT plots (Table 2; Figure 2b), and the amount of leaf litter decomposing over the soil surface did not differ between T and NT plots. Litter C and N percentages were also similar across leaf litter and root litter inputs (Table 2), without changes in C:N ratios across treatments (Figure 2c).

**Table 2:** Results of the ANOVAs on soil and microbial fractions and root and leaf litter variables.

**Factor**	**Trenching**	**Leaf litter input**	**Depth**	Trenching*Leaf litter input	Trenching*Depth	Leaf litter input*Depth	Trenching*Leaf litter input*Depth
DOC	0.76	12.39^***^	25.21^***^	1.31	1.21	0.3	0.08
C_micro_	14.27^***^	1.12	105.21^***^	1.01	0.09	0.2	0.12
SOC	0.01	0.76	77.34^***^	0.1	0.24	0.05	0.31
TN	0.08	0.69	106.18^***^	0.22	0.05	0.03	0.25
C:N_soil_	0.58	0.51	12.50^***^	0.34	1.28	0.82	0.02
ρ_soil_	0.36	0.42	0.01	0.05	0.27	0.03	0.19
Dry weight roots	7.27^**^	0.03	10.11^**^	0.18	0.88	1.13	1.22
Dry weight litter	2.48	30.38^***^		0.77			
C_litter_	0.00	0.56		0.92			
N_litter_	1.81	0.15		0.04			
C:N_litter_	3.53	0.44		3.32			
18:2ω6,9 PLFA	7.25^*^	0.4		1.06			
Bacterial PLFAs	2.01	0.8		0.22			
Fungal:bacterial PLFAs	8.35^**^	1.00		0.22			
Total PLFAs	7.10^*^	0.01		1.48			
Relative EcM abundance	59.55^***^	1.21		4.30^*^			
Relative Saprotrophs abundance	12.13^**^	0.38		3.43^*^			
Contact-distances EcM		1.27					
Short-distances EcM		1.97					
Short-medium-distances EcM		4.23^*^					
Medium-distances EcM		0.55					
Long distances EcM		0.63					

*Values of the F statistic are presented. The effects of trenching, leaf litter inputs, soil depth, and their interactions are shown. The effect of soil depth is not applicable for litter variables. The effect of soil depth and trenching is not applicable for ectomycorrhiza exploration types, since the root trenching suppressed virtually all ectomycorrhizal fungi. DOC, Dissolved organic carbon; C_micro_, microbial carbon; SOC, soil organic carbon; TN, total soil nitrogen; C:N_soil_, soil C:N ratios; ρ_soil_, soil bulk density; C_litter_, carbon concentration in litter; N_litter_, nitrogen concentration in litter; C:N_litter_, C:N ratios in litter; PLFAs, phospholipid fatty acids. Statistically significant effects are marked in bold*.

* *0.01 < P ≤ 0.05*;

** *0.001 < P ≤ 0.01*;

*** *P ≤ 0.001*.

Microbial biomass was consistently higher in NT plots across soil depths (0–5 and 5–10 cm), but it was not affected significantly by the amount of litter addition (Table 2; Figure 2d). On the contrary, the soil DOC increased consistently with the amount of leaf litter inputs, but not with the presence of roots (Table 2; Figure 2e). Neither the amount of leaf litter nor the root exclusion by trenching provoked significant changes in SOC, soil TN, C:N ratios or soil bulk density 3 years after the experiment establishment (Table 2; Figure 2f), and therefore, treatments did not affect soil C and N stocks significantly either. DOC, microbial biomass C, SOC, and TN decreased consistently with soil depth (Table 2), while the soil C:N ratios showed an increase at the sub-superficial 5–15 cm depth (Table 1).

### Fungal Community Shifts

**Figure 3:**
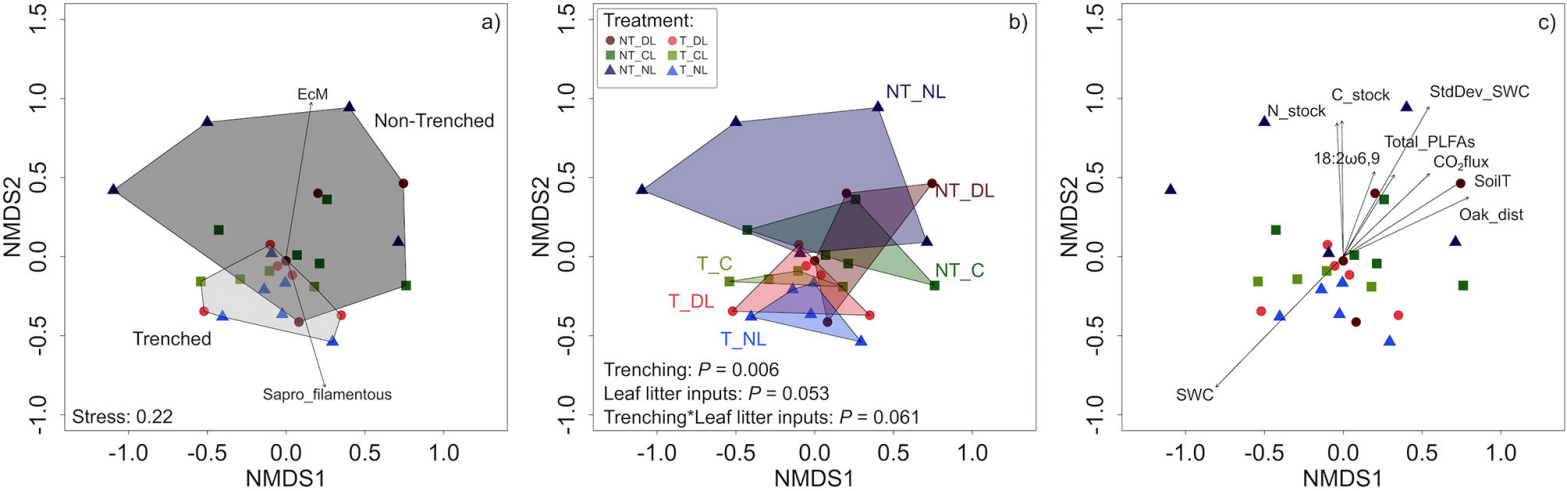
Non-metric multidimensional matrix of soil fungal communities in the trenching and leaf litter inputs treatments. **(a)** Trenching levels are indicated by shaded polygons and relative abundances of fungal trophic types represented by vectors. **(b)** Leaf litter levels are indicated by different colored polygons. **(c)** Gradient direction and strength of environmental variables indicated by vectors. Only variables with a *P* < 0.05 are shown. EcM, ectomycorrhiza abundance; Sapro_filamentous, filamentous saprotroph abundance; SWC, soil moisture; N_stock, soil N stock; C_stock, soil C stock; 18:2ω6,9, 18:2ω6,9 PLFA; StdDev_SWC, soil moisture standard deviation; Total_PLFAs, Total PLFAs; CO_2_flux, CO_2_ flux; SoilT, soil temperature; Oak_dist, distance to the closest oak tree; NT_DL, Non-Trenched and Doubled Litter; NT_CL, Non-Trenched and Control Litter; NT_NL, Non-Trenched and No-Litter; T_DL, Trenched and Doubled Litter; T_CL, Trenched and Control Litter; T_NL, Trenched and No-Litter.

The root exclusion by trenching altered the fungal communities present in the upper 5 cm of mineral soil and drastically reduced the proportion of EcM fungi DNA reads (Table 3; Figure 3a). By contrast, T plots showed a higher proportion of saprotrophs, particularly of filamentous fungi. The amount of leaf litter inputs also altered the composition of soil fungal communities (Figure 3b), with a marginal interaction between root and leaf litter inputs. Soil moisture was higher and less variable (lower standard deviation) where roots were excluded (Table 3; Figure 3c). Soil CO_2_ fluxes, soil temperature, fungal and total PLFAs, soil C and N stocks and the distance to the closer oak tree showed an increasing gradient in the direction of NT plots. Fungal communities were also more heterogeneous (higher pair-wise dissimilarities) in presence of roots than when roots were excluded (*F* = 2.09, *P* = 0.001, Figure 3b), while dissimilarities were not altered by litter inputs. Nonetheless, dissimilarity indices showed highest values in the combined NT and NL plots.

**Table 3:** Regression weights of the variables fitted in the NMDS analysis in Figure 3c.

**Category**	**Variable**	**NMDS1**	**NMDS2**	***r^**2**^***	***P***
Trophic type	Animal parasite	−0.710	−0.704	0.089	0.313
	Plant pathogen	−0.587	−0.809	0.033	0.628
	Mycoparasite	−0.666	−0.746	0.090	0.287
	Sapro Brown rot	0.312	0.950	0.025	0.679
	Sapro Filamentous	0.287	−0.958	0.445	**0.002**
	Sapro White rot	−0.907	−0.422	0.103	0.263
	Sapro Yeast	−0.930	−0.368	0.023	0.751
	Arbuscular mycorrhiza	−0.481	−0.877	0.019	0.803
	Ectomycorrhiza	0.163	0.987	0.587	**0.001**
Soil and environmental variables	Soil bulk density	0.47735	0.87871	0.0891	0.317
	% Stones	0.85290	−0.52207	0.0360	0.610
	Inorganic C	0.78558	−0.61876	0.0077	0.901
	Distance to closer oak	0.90257	0.43055	0.3629	**0.003**
	Distance to closer beech	−0.92891	−0.37031	0.0643	0.448
	Root biomass	0.23218	0.97267	0.1468	0.138
	Soil moisture	−0.70474	−0.70947	0.6324	**0.001**
	Soil moisture S.D.	0.51806	0.85534	0.5492	**0.001**
	Soil temperature	0.85110	0.52500	0.3300	**0.011**
	Soil temperature S.D.	−0.60766	−0.79420	0.0430	0.595
	Soil CO_2_ flux	0.72530	0.68843	0.2739	**0.025**
	Litter dry weight	0.64275	−0.76608	0.0341	0.644
	Litter C	0.58819	−0.80872	0.0484	0.552
	Litter N	0.61027	−0.79219	0.0435	0.586
	Litter C:N ratio	0.65895	−0.75219	0.0435	0.590
	SOC	−0.86350	−0.50435	0.1256	0.206
	TN	−0.91565	−0.40198	0.0634	0.445
	Soil C:N ratio	−0.78242	−0.62276	0.3075	0.052
	DOC	−0.61375	−0.78950	0.0730	0.372
	Microbial biomass C	0.33463	0.94235	0.1121	0.237
	Soil C stock	−0.01278	0.99992	0.3497	**0.008**
	Soil N stock	−0.04971	0.99876	0.3396	**0.009**
	C18:2ω6,9 PLFA	0.43539	0.90024	0.2387	**0.050**
	Total PLFAs	0.57168	0.82047	0.2384	**0.040**

*r^2^, Squared Pearson correlation; P, Critical probability of the analysis. Statistically significant effects are marked in bold*.

**Figure 4:**
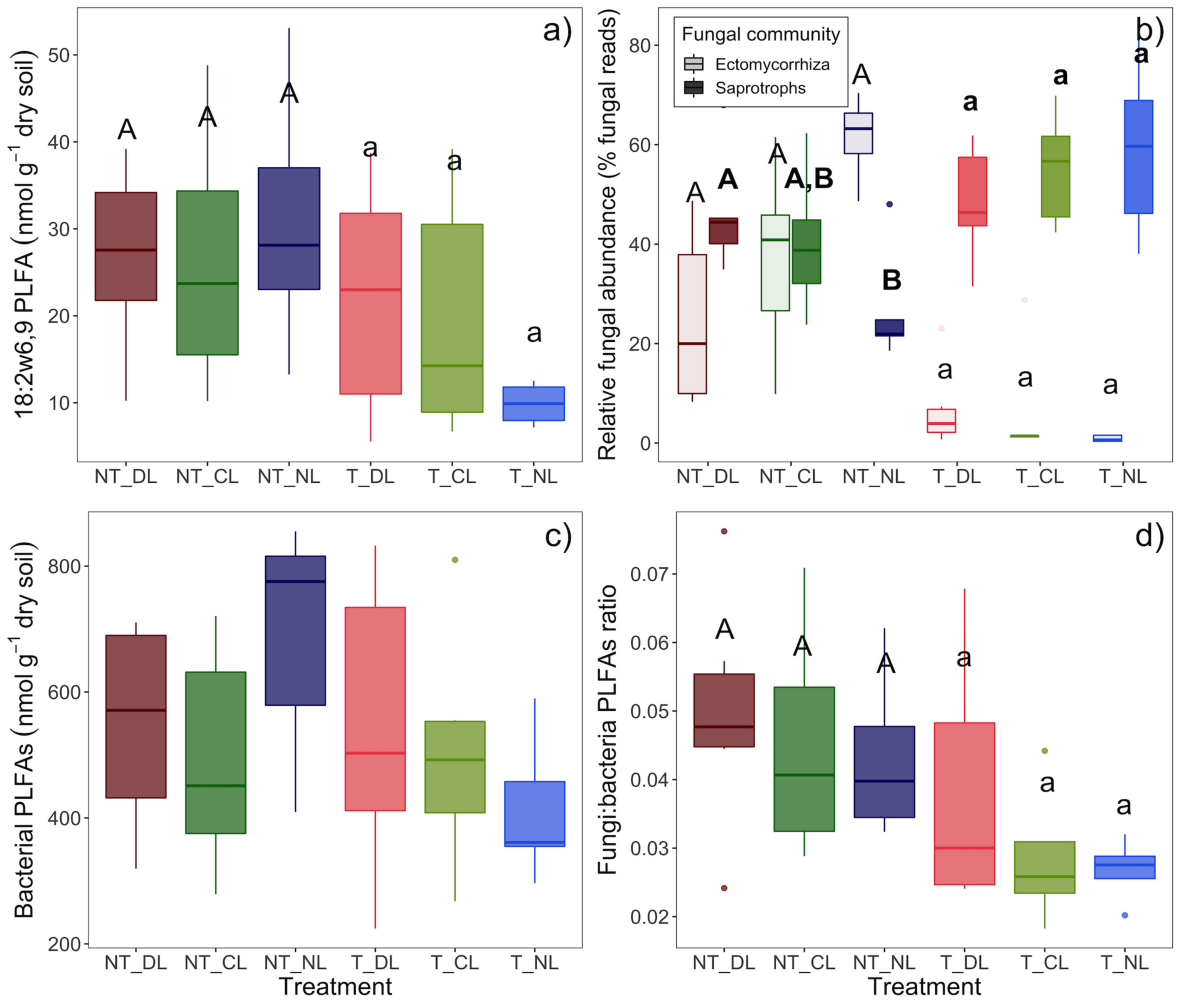
Abundance estimations of soil microbial groups in the trenching and leaf litter inputs treatments. **(a)** 18:2ω6,9 PLFA marker (fungal abundance), **(b)** relative abundances of saprotrophic and ectomycorrhizal fungi (% of fungal DNA reads), **(c)** sum of bacterial PLFAs, **(d)** ratio of fungal to bacterial PLFAs. Box-plots represent the values of *n* = 5 plots per treatment. Different letter case indicates significant effect of trenching according two-ways ANOVA, different letters indicate significant differences among leaf litter input levels within each trenching level according one-ways ANOVA. In **(b)**, bold letters indicate treatment effects on saprotrophic fungi and normal letters indicate treatment effects on ectomycorrhizal fungi. NT_DL, Non-Trenched and Doubled Litter; NT_CL, Non-Trenched and Control Litter; NT_NL, Non-Trenched and No-Litter; T_DL, Trenched and Doubled Litter; T_CL, Trenched and Control Litter; T_NL, Trenched and No-Litter.

In accordance with microbial biomass, fungal PLFA 18:2ω6,9, total PLFAs and fungal to bacterial PLFA ratios were higher at NT plots (Table 2; Figures 4a,d) as well as Actinobacteria (*P* = 0.0264), while effect of leaf litter inputs on these PLFA groups was not statistically significant. Bacterial PLFA, by contrast, was not affected by the trenching or litter addition treatments (Figure 4c), and the same was true for Gram positive and Gram negative bacteria separately, and for Eukariota microbial communities.

**Figure 5:**
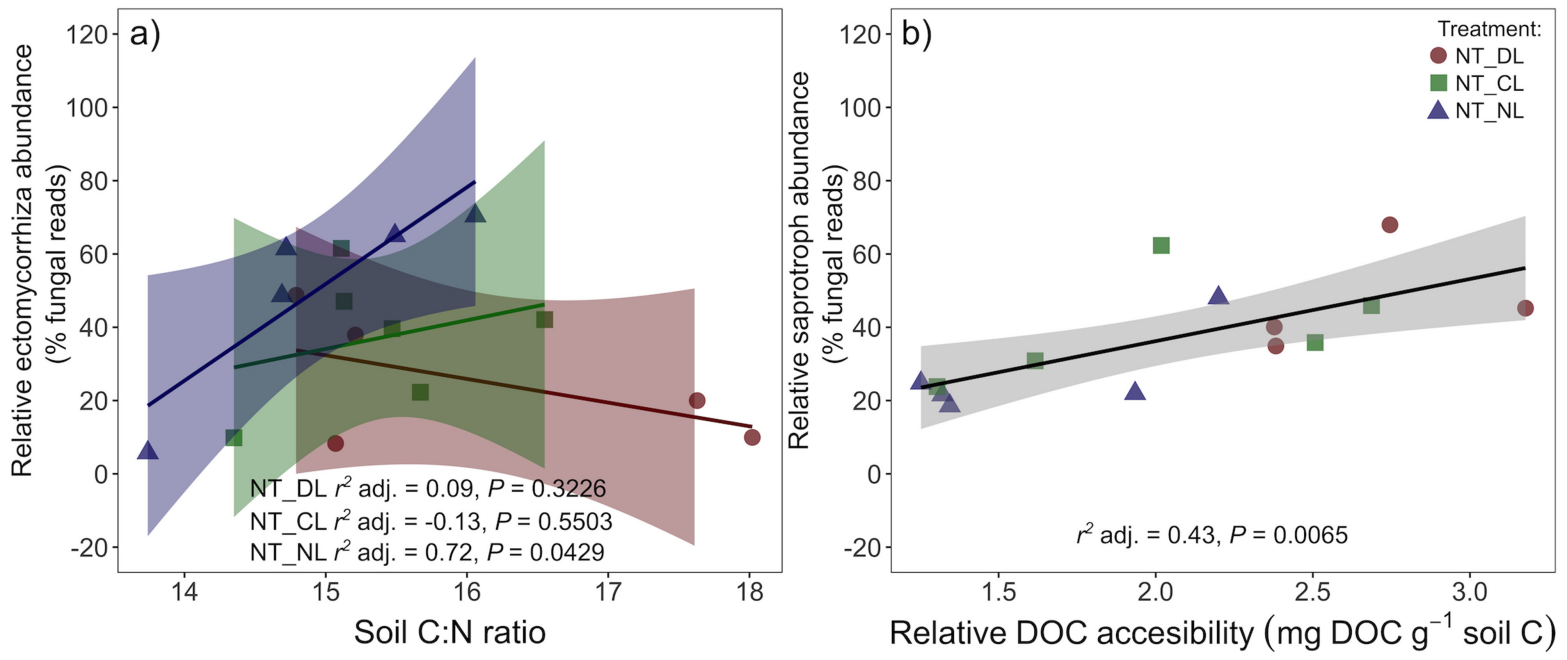
Correlation between the **(a)** relative abundance of ectomycorrhizal fungi and the soil C:N ratios in the different leaf litter input treatments in Non-Trenched plots and **(b)** the relative abundance of saprotroph fungi and the relative accessibility of the soil DOC pool in Non-Trenched plots. The relative accessibility of the DOC pool was calculated as the ratio of DOC to the SOC pool in soil. *r*^2^ adj. = Adjusted Pearson correlation. Shaded areas indicate the 95% confidence intervals. NT_DL, Non-Trenched and Doubled Litter; NT_CL, Non-Trenched and Control Litter; NT_NL, Non-Trenched and No-Litter; T_DL, Trenched and Doubled Litter; T_CL, Trenched and Control Litter; T_NL, Trenched and No-Litter.

The reduction of EcM fungi by root trenching is also evidenced by a 94–73% decrease in their relative abundances (Table 2; Figure 4b). Overall, relative EcM abundances were not significantly affected by the leaf litter inputs, but they increased in NT plots with decreasing leaf litter inputs, causing a significant effect of leaf litter inputs in NT plots (*P* = 0.0346) and a significant Trenching^*^Leaf litter input interaction (*P* = 0.0248). EcM relative abundance in NT plots was positively correlated with soil C:N ratios in absence of leaf litter inputs (NL, Figure 5a), whereas the slopes of this correlation decreased progressively with the amount of leaf litter inputs (CL and DL). Contrary to the relative EcM abundances, the relative abundances of saprotrophic fungi increased in T plots. Leaf litter inputs also did not have an overall effect on saprotrophs relative abundances, but they increased with leaf litter inputs in NT plots, causing a marginal leaf litter effect on NT plots (*P* = 0.0814) and a significant Trenching^*^Leaf litter input interaction (*P* = 0.0474, Figure 4b). Saprotroph relative abundance in NT plots was, moreover, positively correlated with the DOC accessibility in soil relative to total SOC (Figure 5b).

**Figure 6:**
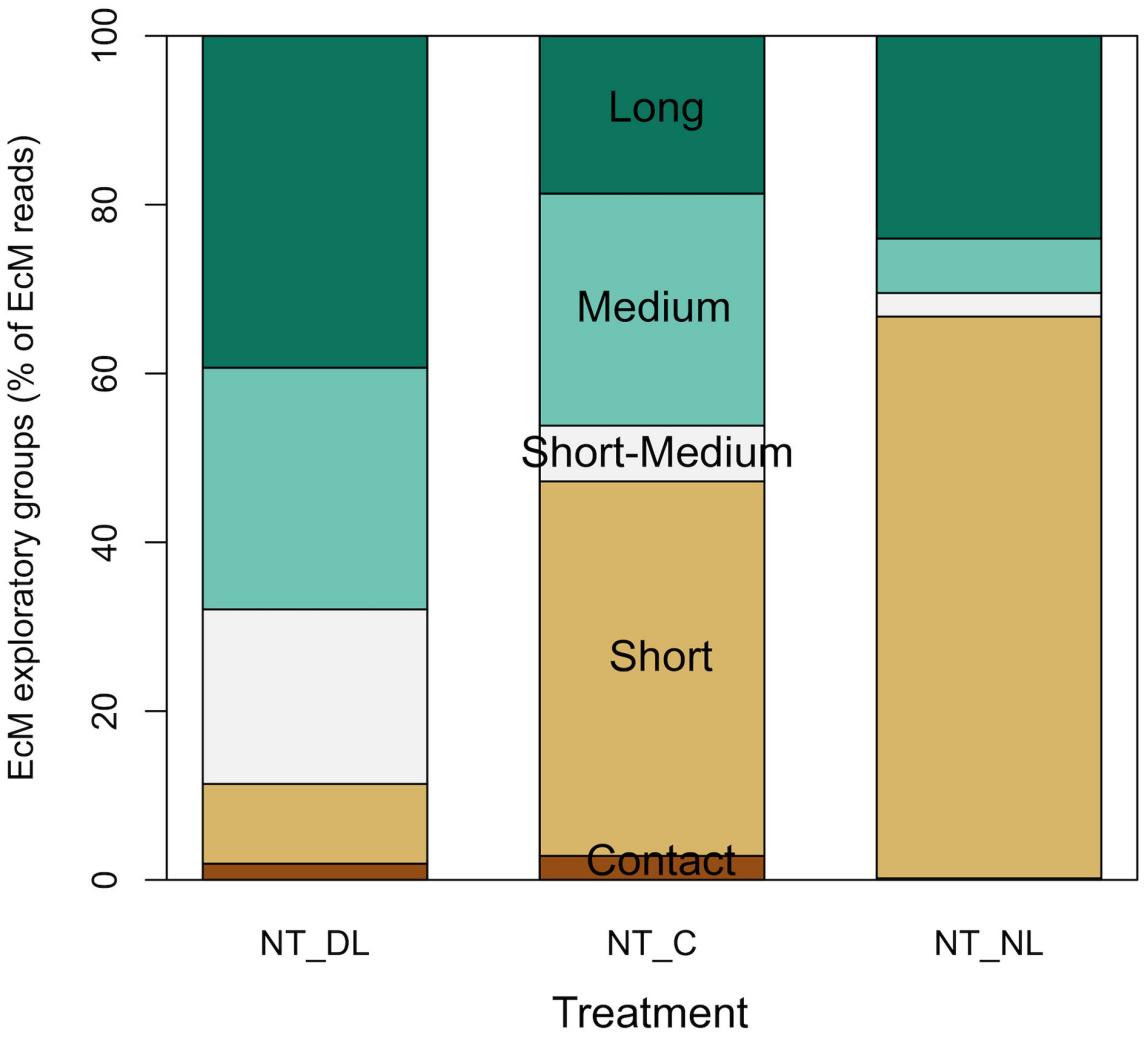
Shifts in the relative abundances of ectomycorrhizal fungi groups of different exploratory strategies for each leaf litter input treatment. Only abundances of ectomycorrhizal fungi in Non-Trenched plots are presented, since the root trenching suppressed virtually all ectomycorrhizal fungi. NT_DL, Non-Trenched and Doubled Litter; NT_CL, Non-Trenched and Control Litter; NT_NL, Non-Trenched and No-Litter; T_DL, Trenched and Doubled Litter; T_CL, Trenched and Control Litter; T_NL, Trenched and No-Litter.

The amount of leaf litter inputs also determined the presence of EcM fungal communities with different exploration strategies in NT plots (Figure 6), although the effect of leaf litter input was not significant in all the exploration types due to the high variability among blocks (Table 2). Short-medium, medium and long-distances exploration types of EcM fungi tended to dominate at doubled leaf litter inputs (*P* = 0.0939 for the sum of these exploration types and *P* = 0.0406 for short-medium distances), while short-distance exploration strategies tended to be more abundant, although not significantly, in absence of leaf litter inputs.

The high variability in the relative abundance of fungal groups among blocks and within treatments was associated, besides to spatial variability of soil properties and microclimate, to the differences in the distances from roots of the different tree species (oak and beech). We detected positive correlations between the distance to the nearest oak tree and the relative abundance of ectomycorrhizal fungi (*P* = 0.0034, *R*^2^ = 0.7032) and negative correlations with the relative abundances of plant pathogen (*P* = 0.0076, *R*^2^ = 0.6584) and arbuscular mycorrhizal fungi (*P* = 0.056, *R*^2^ = 0.6767) in NT plots. In the same plots, there were also positive correlations between the distances to the nearest beech tree and the relative abundances of arbuscular mycorrhiza (*P* = 0.0016, *R*^2^ = 0.7391) and plant pathogen fungi (*P* = 0.0007, *R*^2^ = 0.7723).

### Soil CO_2_ Fluxes

**Figure 7:**
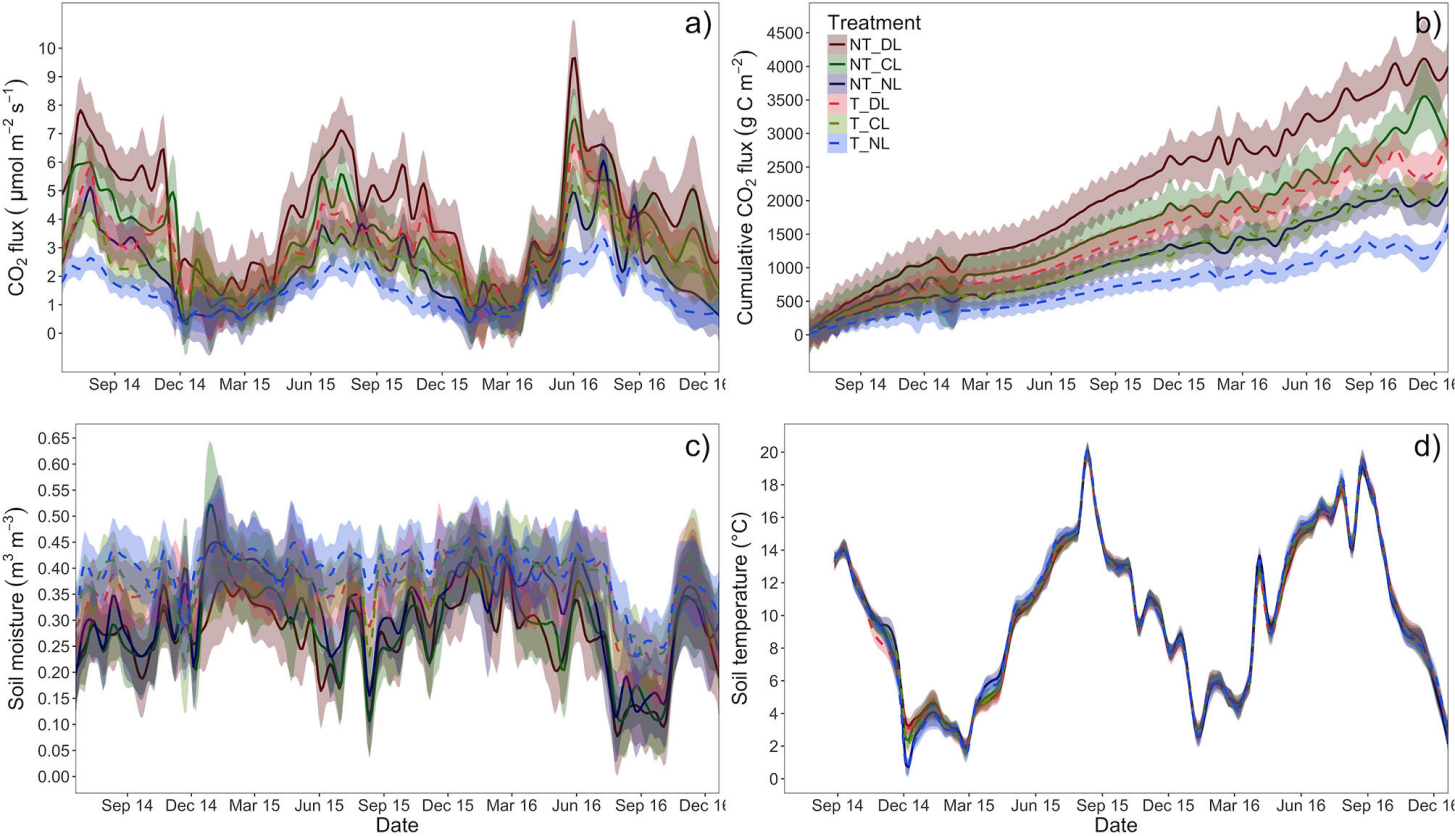
**(a)** Time course of soil CO_2_ fluxes, **(b)** cumulative soil CO_2_ fluxes, **(c)** soil moisture, and **(d)** soil temperature in each of the trenching and leaf litter input treatments. Lines represent the loess smooth of *n* = 5 per treatment and date. Shadowed areas around lines represent the 95% confidence interval of the mean. NT_DL, Non-Trenched and Doubled Litter; NT_CL, Non-Trenched and Control Litter; NT_NL, Non-Trenched and No-Litter; T_DL, Trenched and Doubled Litter; T_CL, Trenched and Control Litter; T_NL, Trenched and No-Litter.

**Figure 8:**
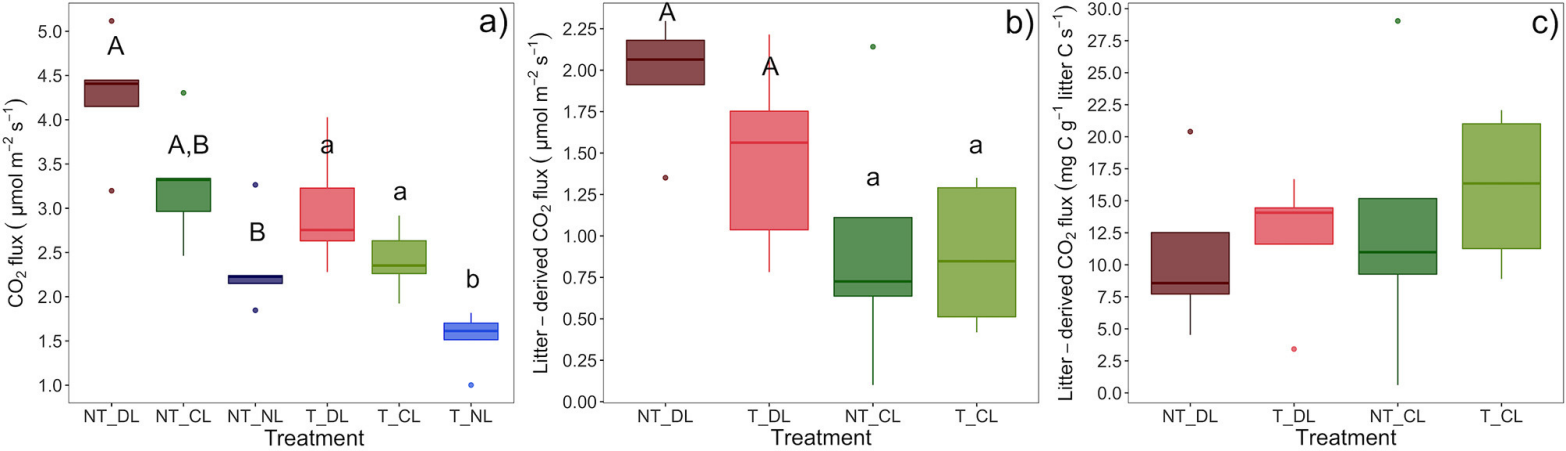
**(a)** Mean CO_2_ flux, **(b)** CO_2_ flux excess associated to the leaf litter inputs taking the No-Litter treatment as a baseline in each of the trenching and leaf litter input treatments and **(c)** CO_2_ flux excess associated to the leaf litter inputs per unit of leaf litter carbon. Box-plots represent the values of *n* = 5 plots per treatment. Different letter case indicates significant effect of trenching **(a)** or litter **(b)** according two-ways ANOVAs. Different letters indicate significant differences among leaf litter inputs **(a)** or trenching levels **(b)** within each level of the other factor. NT_DL, Non-Trenched and Doubled Litter; NT_CL, Non-Trenched and Control Litter; NT_NL, Non-Trenched and No-Litter; T_DL, Trenched and Doubled Litter; T_CL, Trenched and Control Litter; T_NL, Trenched and No-Litter.

Soil CO_2_ fluxes were consistently highest in NT and increased with leaf litter inputs (DL > CL > NL) (Table 4; Figures 7a, 8a), without significant interactions between trenching and leaf litter inputs. Nonetheless, the excess of CO_2_ flux associated to litter inputs did not differ significantly between T and NT (Figure 8b) and differences between litter input levels also disappeared when fluxes were standardized per unit of leaf litter C, although fluxes were slightly lower in NT plots (Figure 8c). Soil CO_2_ fluxes also varied with time (Table 4), with a lower contribution from roots during winter and a higher contribution from leaf litter from early summer to autumn (Figures 7a,b).

**Table 4:** Summary of Repeated Measures Analysis of Variance (rmANOVA) for the monthly means of soil CO_2_ fluxes, soil temperature, and soil moisture.

**Source**	**Soil CO**_****2****_ **flux**			**Soil temperature**			**Soil moisture**		
**Between-subject**	**df**	***F***	***P***	**df**	***F***	***P***	**df**	***F***	***P***
Trenching	1, 24	19.97	0.0002	1, 24	0.02	0.8813	1, 24	21.43	<0.0001
Leaf litter input	2, 24	29.35	<0.0001	2, 24	0.52	0.6001	2, 24	1.41	0.2634
Trenching*Leaf litter input	2, 24	0.22	0.8061	2, 24	0.99	0.3842	2, 24	0.06	0.9386
**Within-subject**	**df**	***F***	***P***	**df**	***F***	***P***	**df**	***F***	***P***
Date	29, 696	152.47	<0.0001	26, 624	1045.36	<0.0001	29, 696	42.52	<0.0001
Date*Trenching	29, 696	2.08	0.0008	26, 624	0.60	0.9416	29, 696	6.07	<0.0001
Date*Leaf litter input	58, 696	4.14	<0.0001	52, 624	1.01	0.4613	58, 696	0.65	0.9783
Date*Trenching*Leaf litter input	58, 696	0.96	0.5684	52, 624	0.12	1.0000	58, 696	0.67	0.9699

*df = degrees of freedom of the numerator and denominator, respectively. F = Value of the F statistic, according to unadjusted univariate F-tests for contrasts within subjects. P = Critical probability of the analysis*.

Soil temperature in the upper 10 cm of soil was not affected by the presence of roots or the amount of litter inputs to the soil (Table 4; Figure 7c). By contrast, soil moisture was lowest in NT plots, where roots were present (Table 4; Figure 7d), particularly during the growing season.

## Discussion

The ecological relations between EcM and saprotroph fungi can determine the fate of SOM and soil C stabilization (Lindahl and Tunlid, 2015; Wang et al., 2017). The amount of aboveground litter inputs modulates the availability and accessibility of organic substrates in soil. We present evidences suggesting that this generates ecological niches for different fungal trophic types (saprotrophs vs. EcM fungi) and EcM lineages (EcM exploration types) that are favored according to their resource demands and acquisition strategies, determining their abundances. As a result, substrate availability and accessibility may modulate the ecological relations between EcM and saprotrophic fungal communities and, ultimately, the relative role of EcM vs. saprotroph fungi on litter decay and SOM transformation.

### Mechanisms Modulating the ecological Relations Between EcM and Saprotrophic Fungi

Fungal communities in soil are largely structured by the availability of limiting substrates (Waldrop et al., 2006; Cline and Zak, 2015), among other factors. We hypothesized that the amount of leaf litter inputs, which generates a range of availability and accessibility of organic substrates in soil, determines the ecological interactions between EcM and saprotroph fungi and creates diverse niches in which the most adapted fungal guilds are selected according to their resource demands and acquisition strategies. Increasing amounts of litter inputs certainly created a gradient of dissolved organic C in the upper 10 cm of soil, both in Trenched and Non-Trenched plots (Table 2; Figure 2e), although soil organic C, total soil N concentrations and C:N ratios did not show significant changes after 3 years of altered root and leaf litter inputs (Table 2; Figure 2f). The root exclusion succeeded in suppressing virtually all ectomycorrhizal fungi (Figure 4b), contributing to the decrease in the total microbial biomass C, fungal and total PLFAs in soil (Table 2; Figures 2d, 4a). Microbial biomass C and fungal PLFAs however did not change in response to the DOC gradient created in soil by the litter manipulation, but the relative abundances of EcM and saprotroph fungi shifted with leaf litter inputs in Non-Trenched plots (Figures 3b, 4b). The amount of litter inputs therefore selected different fungal communities according to their resources demands and acquisition strategies, altering the abundances of saprotrophic and EcM fungi.

Saprotrophs increased with leaf litter inputs and with soil DOC accessibility in Non-Trenched plots (Figure 5b), which evidences the higher reliance of saprotrophs on fresh and accessible organic substrates compared to EcM fungi. On the other hand, EcM fungal abundance increased progressively with decreasing leaf litter inputs, and they proliferated more at higher soil C:N ratios in absence of leaf litter inputs (NL, Figure 5a). Conditions of limited access to organic substrates in NL resulted advantageous for EcM, where they resulted better competitors at lower organic matter quality. Declines in EcM fruiting bodies, EcM species richness and dominance have been reported in response to increases in the litter layer and N deposition in Europe and US (Arnolds, 1991; Peter et al., 2001; Averill et al., 2018; Jo et al., 2019). The removal of litter and humus layer have been actually used as practices to restore EcM fungal diversity (Baar and Kuyper, 1998; Smit et al., 2003). Trees generally reduce the belowground C allocation to EcM fungi with increasing N availability at regional or larger scales (Phillips et al., 2011). At the moderate levels of soil N availability in our study, low decomposition and N mineralization rates in the litter exclusion treatment may have acted as a local stress signal for the trees, increasing root elongation, exudation, and the C supply to EcM fungi to enhance N acquisition in exchange (Drew, 1975; Hodge, 2005).

The direct access to plant host sugars may also facilitate EcM fungi an efficient interception and immobilization of N leaching through the soil (Pena et al., 2013) and the production of extracellular peroxidases needed to acquire the N sequestered in complex recalcitrant compounds (Smith and Read, 2008; Bödeker et al., 2009; Lindahl and Tunlid, 2015). Accordingly, Steidinger et al. (2019) have shown that EcM trees dominate in climatic areas of low decomposition rates. Moreover, the amount C transferred from roots to EcM fungi have shown to decrease drastically in presence of saprotroph wood-decomposing fungi (Leake et al., 2001). It is therefore likely that litter inputs, organic matter quality and nutrient availability interact to select functionally distinct fungi, affecting in turn, plant nutrition and C allocation.

Our results point to the emergence of divergent specialization strategies to the available substrates, but we were not able to detect unequivocal signs of competition between saprotrophs and EcM fungi for common resources. Despite the relative abundance of saprotroph fungi increased when EcM fungi were excluded in Trenched plots (Figure 4b), trenching also decreased the total fungal abundance (Figure 4a), precluding to detect significant changes in the absolute abundances of saprotrophs between T and NT plots. The proportion of unassigned OTUs may have had some contribution on the lack of unequivocal signs of competition between ectomycorrhizal and saprotrophic fungi. The proportion of unassigned OTUs was higher in Trenched plots (Supplementary Figure 3, *P* = 0.003), which most likely belong to saprotrophic fungi, since EcM fungi are fairly well-known and well-represented in the DNA databases. Nonetheless, our data do not show a clear deceleration of decomposition rates in presence of EcM fungi. The litter-derived CO_2_ fluxes per unit of litter C did not differ significantly between Trenched (without EcM fungi) and Non-Trenched plots (with EcM fungi) (Figure 8c). If any, the inhibiting effect of EcM fungi on litter decomposition rates was very low to be detected in this case, considering that lower levels of soil moisture in presence of roots (Table 4; Figure 7d) may have also contributed to underestimate the litter-associated excess CO_2_ flux in Non-Trenched plots. Smith and Wan, 2019 have recently found that ectomycorrhizal fungi slow leaf litter decomposition only in forests where litter inputs are highly recalcitrant. In agreement to that, soil C:N ratios in our study (Table 1) were relatively low compared to other studies where a negative effect of EcM fungi on litter decomposition rates have been reported (Gadgil and Gadgil, 1971, 1975; Koide and Wu, 2003; C:N = 26 and C:N = 43.6, respectively). Higher soil temperatures may have also contributed to faster microbial mineralization rates and N release from SOM in our case. Higher N availability in these soils and the specialization of EcM and saprotroph fungi on divergent target substrates may have allowed them to co-exist without any apparent signs of competition. In support to that, Kyaschenko et al. (2017) found a higher proliferation of saprotrophs, oxidative enzymatic activities and N cycling in fertile sites with higher productivity and N availability, whereas EcM fungi suppressed enzymatic oxidation in less fertile soils. Similarly, Sterkenburg et al. (2018) showed that the effect of EcM fungi on decomposition rates changed across stages of SOM decomposition, where EcM fungi slow down litter decomposition at the soil surface but accelerate SOM decomposition at deeper soil layers. Taken together, these results support the idea that the influence of EcM fungi on litter and SOM decay is strongly context dependent (Fernandez and Kennedy, 2015; Zak et al., 2019), where N availability, SOM quality and the accessibility of organic substrates are key factors that will determine the structure of the soil fungal community, their ecological relations and the net effect of EcM fungi on SOM transformation.

### Shifts in Exploration Types of EcM Fungi

EcM fungal communities were not only more successful when organic substrates were scarce, but they can also optimize resource acquisition through different exploration strategies according to available substrates (Agerer, 2006; Tedersoo and Smith, 2013). Short-distances exploration types of EcM fungi tended to dominate in the litter exclusion treatment (Figure 6), although the effect was not statistically significant. These EcM guilds are argued to predominantly utilize simple N forms or byproducts from advanced stages of decomposition (ammonium, nitrate and simple aminoacids, Hobbie and Agerer, 2010). Short-distances EcM fungi also invest lower amounts of C on hyphae elongation, which represents a carbon-conservative strategy that may make them more competitive in areas with higher EcM fungal density (Peay et al., 2011). By contrast, shifts to a higher abundance of long, medium and short-medium-distance exploration types at increasing litter inputs suggests a shift in the target substrate. Long-distance EcM guilds utilize widely dispersed, spatially concentrated resources and have higher capacity of SOM oxidation and organic nitrogen uptake (Hobbie and Agerer, 2010; Lilleskov et al., 2011, Nicolás et al., 2019). Despite the high variability among replicate blocks in our study did not allowed to support this hypothesis, it may be possible that increasing levels of leaf litter inputs enhance the proliferation of EcM fungal groups with the ability to exploit organic N substrates at larger distances from the plant root, such as the decomposing litter (Rineau et al., 2013). These results represent an open door to future research on the role that different exploration types of EcM fungi may have on litter decomposition.

## Conclusions

The ecological interaction between saprotroph and EcM fungal communities can have crucial implications for organic matter decomposition and soil C stabilization. Our results show the proliferation of saprotrophs and EcM fungi of different exploration types based on their acquisition strategies and the available target substrates. The amount of leaf litter inputs, and consequently, substrate availability and accessibility, modulated the abundances of these fungal groups and their ecological relations. EcM fungi were generally favored at low levels of leaf litter inputs and lower SOM quality, where short-distances exploration types may be more competitive, whereas saprotrophs and longer exploration types of EcM fungi tended to dominate at high levels of leaf litter inputs and accessibility of labile organic substrates. These patterns, if confirmed in further studies, may have important implications for decomposition rates in the face of global driven changes in plant allocation patterns. Accordingly, saprotroph fungi may have a key contribution on the breakdown of organic substrates at increasing levels of aboveground tree productivity and leaf litter inputs, whereas the role of EcM fungi on enhancing plant N acquisition may become increasingly relevant at low levels of plant aboveground litter inputs. These results also represent a path forward for future experiments testing the role of different EcM exploration types of fungi on litter and SOM decomposition rates.

## Data Availability Statement

The datasets presented in this study can be found in online repositories. The names of the repository/repositories and accession number(s) can be found at: Sequence Read Archive (SRA, http://www.ncbi.nlm.nih.gov/bioproject/747155, BioProject ID PRJNA74715). Data on soil variables and CO_2_ fluxes are archived and publicly available at the online archives of the Helmholtz Centre for Environmental Research GmbH - UFZ (https://www.ufz.de/record/dmp/archive/7013/de/ and https://www.ufz.de/record/dmp/archive/7016/de/, respectively).

## Author Contributions

SM-J, CR, and MC conceived the research. SM-J, DR, EV, AR, MS, and CR contributed data. SM-J conducted analyses, generated figures, and prepared the original draft. All authors provided comments on the manuscript.

## Conflict of Interest

The authors declare that the research was conducted in the absence of any commercial or financial relationships that could be construed as a potential conflict of interest.
