# Supplementary material for: Shifts in the Abundances of Saprotrophic and Ectomycorrhizal Fungi With Altered Leaf Litter Inputs
Source: Front Plant Sci. 2021 Jul 21;12:682142. doi: 10.3389/fpls.2021.682142 (PMC8336600; doi:10.3389/fpls.2021.682142)
Supplement: Supplementary file 1 [file Data_Sheet_1.DOCX]

## Supporting Information

Article title: **Shifts on the abundances of saprotrophic and ectomycorrhizal fungi at altered leaf litter inputs**

Authors: Marañón-Jiménez S., Radujkovic D., Verbruggen E., Grau O., Cuntz M., Peñuelas J., Richter A., Schrumpf M., Rebmann C.

The following Supporting Information is available for this article:

**Fig. S1** a) Rarefaction curves for each of the samples. b) OTUs accumulation curves.

**Fig. S2** Non-metric multidimensional matrix of soil fungal communities in the trenching and litter inputs treatments using non-rarefied relative fungal abundances.

**Fig. S3** Shifts in the relative abundances of each fungal trophic type in each treatment. **Supplementary file:** Identification of OTUs, assignments as trophic type and exploratory groups of ectomycorrhiza and relative abundance of the OTUs in each of the samples.

**Fig. S1** a) Rarefaction curves for each of the samples. Horizontal lines represent the rarefied OTUs richness of each sample. The vertical line represents the size of the smallest sample. b) OTUs accumulation curves. Vertical lines represent the standard deviation of the expected OTUs richness. NT_DL: Non-Trenched and Doubled Litter, NT_CL: Non-Trenched and Control Litter, NT_NL: Non-Trenched and No-Litter, T_DL: Trenched and Doubled Litter, T_CL: Trenched and Control Litter, T_NL: Trenched and No-Litter.

**Fig. S2** Non-metric multidimensional matrix of soil fungal communities in the trenching and leaf litter inputs treatments using non-rarefied relative fungal abundances. a) Trenching levels are indicated by shaded polygons, b) Leaf litter levels are indicated by different colored polygons. NT_DL: Non-Trenched and Doubled Litter, NT_CL: Non-Trenched and Control Litter, NT_NL: Non-Trenched and No-Litter, T_DL: Trenched and Doubled Litter, T_CL: Trenched and Control Litter, T_NL: Trenched and No-Litter.

**Fig. S3** Shifts in the relative abundances of each fungal trophic type in each treatment. The relative abundance of unassigned OTUs is also presented. NT_DL: Non-Trenched and Doubled Litter, NT_CL: Non-Trenched and Control Litter, NT_NL: Non-Trenched and No-Litter, T_DL: Trenched and Doubled Litter, T_CL: Trenched and Control Litter, T_NL: Trenched and No-Litter.

**Supplementary file:** Identification of OTUs, assignments as trophic type and exploratory groups of ectomycorrhiza (rows) and relative abundance of the OTUs in each of the samples. Samples are labeled in column names with the name of the treatment to which they belong followed by the identification number of replicate. NT_DL: Non-Trenched and Doubled Litter, NT_CL: Non-Trenched and Control Litter, NT_NL: Non-Trenched and No-Litter, T_DL: Trenched and Doubled Litter, T_CL: Trenched and Control Litter, T_NL: Trenched and No-Litter. The percentage of identity (Perc_identity) and the “Expect Value” (E_value, value statistical score which indicates the number of times that an alignment as good or better than that found by Basic Local Alignment Search Tool would be expected to occur by chance, given the size of the database searched) are also included.
